# The assessment of accessory mental foramen in a selected polish population: a CBCT study

**DOI:** 10.1186/s12880-017-0188-6

**Published:** 2017-02-20

**Authors:** Ewa Zmysłowska-Polakowska, Mateusz Radwański, Michał Łęski, Sławomir Ledzion, Monika Łukomska-Szymańska, Michał Polguj

**Affiliations:** 1 Department of Endodontics, Medical University of Lodz, ul. Pomorska 251, Łódź, 92-213 Poland; 2 Department of General Dentistry, Medical University of Lodz, ul. Pomorska 251, Łódź, 92-213 Poland; 3 Department of Angiology, Interfaculty Chair of Anatomy and Histology, Medical University of Lodz, ul. Narutowicza 60, Łódź, 90-136 Poland

**Keywords:** Accessory mental foramen, Cone-beam computed tomography, CBCT, Dental surgery, Endodontics

## Abstract

**Background:**

Accessory mental foramen (AMF) is a rare anatomical variation. When accessory mental foramen is present, the nerves and vessels that go through the mental foramen (MF) must follow alternative courses and special care must be taken during dental treatment planning. The purpose of this study was to evaluate the occurrence and the location of AMF in a selected Polish population using cone-beam computed tomography (CBCT).

**Methods:**

Two hundred CBCT (105 males and 95 females) examinations were evaluated for the presence of AMFs. The location and side of AMFs were reported. The mean distance between MF and AMF was also calculated. The vertical size of MF on the side with and without AMF was measured. The obtained variables were statistically analyzed.

**Results:**

AMFs were observed in 7% of the patients. There was no statistically significant difference between the appearance of AMF and sex (*p* > 0.05). We found no significant difference in the vertical size of MF between individuals with and without AMFs (*p* < 0.05).

**Conclusion:**

Twenty-eight AMFs (7%) were observed from 400 sides of 200 patients. AMFs occurred more often in males (18 AMFs) than in females (10 AMFs). Twenty AMFs (71.4%) were located anteriorly, and eight (28.6%) - posteriorly. Fifteen AMFs (53.6%) were on the right side and thirteen (46.4%) - on the left.

## Background

The mental foramen (MF) is a bilateral opening in the mandible through which nerve endings such as the mental nerve, a branch of the inferior alveolar nerve and corresponding arteries and veins emerge [1–4]. Once the nerve leaves the MF, it branches to innervate the anterior teeth and neighboring structures. The blood vessels supply the soft tissues of the lower jaw [5, 6]. The position of the mental foramen is used as a reference point in the anesthetic technique such as the incisive/mental nerve block. In dental practice, the importance of this structure is mainly related to the positioning of dental implants and to other surgical procedures in this region, for example endodontic surgery. There are some reports on the anatomical variations of the MF such as the presence of accessory mental foramina (AMFs) [2, 5, 7–11]. When AMF is present, the nerves and vessels that go through the mental foramen must follow alternative courses and special care must be taken during dental treatment planning.

Accessory mental foramen has a different description in literature. Some authors described AMF as any additional foramina except the main MF [5, 12]. On the other hand, only those foramina that are integrated with mandibular canal are nominated as AMFs [13–15]. Conversely, the foramen which does not originate in the mandibular canal and its dimensions are relatively small is recognized as a nutrient foramen [16].

The presence of AMF can be evaluated with different methods including macroscopic investigations on dry skulls [14, 17, 18], plane radiographs (including periapical and panoramic views) [19] and computed tomography images (CT or CBCT) [19]. The crucial benefit of cone-beam computed tomography (CBCT) is overcoming the limitations of conventional radiography by producing three-dimensional (3D) images that allow comprehensive evaluation of the anatomy of the chosen region [20]. CBCT is an useful tool that provides detailed information on the structures of the maxillofacial complex, permitting the identification and the evaluation of anatomical variations [19, 20].

The purpose of this study was to evaluate the occurrence and location of the accessory mental foramen in a selected Polish population using CBCT.

## Methods

This retrospective study consisted of 487 CBCTs obtained from 2011 to 2012 in the Radiology Department at the Central Teaching Hospital, Institute of Dentistry, of the Medical University of Lodz (Poland). Images were performed for different diagnostic reasons, such as bone absence for implant placement, assessment of tooth relationships with clinically relevant anatomical structures, dental surgery and diagnosis of radiolucent lesions. The CBCT scans were selected according to the following inclusion criteria: visibility of MF, no lesion observed in the apical area of premolars and MF, no bone resorption occurrence. Only images with availability of precise information about patient age and sex were selected. The exclusion criteria consist of CBCT images with large pathological lesions in mandible and bone fractures in region of examination. Also inadequate picture quality with artefacts caused by osteosynthesis plates/implants or patient movement during exposure were rejected. According to our inclusion and exclusion criteria, the final sample group included data from 200 patients (105 males and 95 females). This study has obtained a positive opinion of the Ethics Committee of Medical University of Lodz, Poland (No. RNN/322/15/KE).

Accessory mental foramen (AMF) in this cross-sectional study was defined as a buccal foramen smaller than the mental foramen and followed by the accessory branch of the mental canal before it exits from the mental foramen, regardless of its location.

**Fig. 1:**
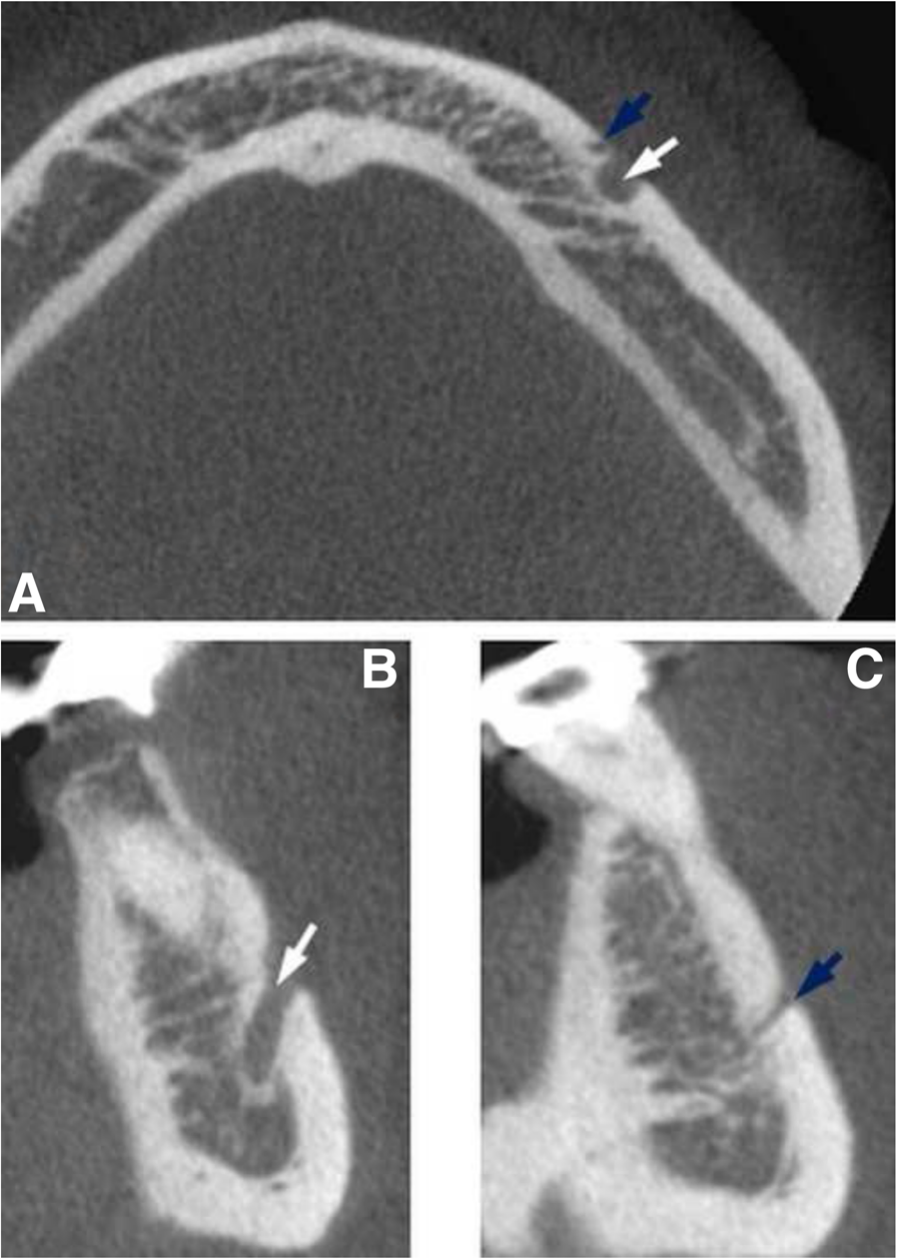
CBCT images of mental and accessory mental foramen. **a** Axial image. **b** Cross-sectional image at the mental foramen. **c** Cross-sectional image at the accessory mental foramen. *White arrowhead* - mental foramen, *blue arrowhead* - accessory mental foramen

**Fig. 2:**
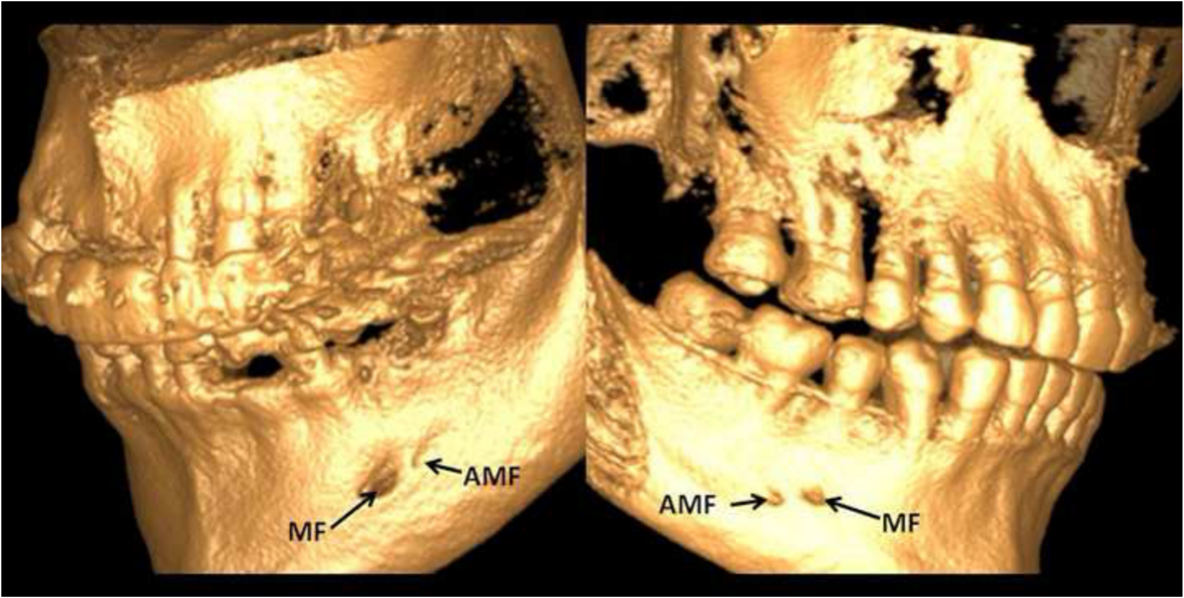
Three-dimensional images (CBCT) of mental foramen and accessory mental foramen

**Fig. 3:**
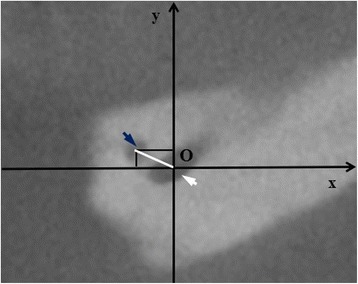
Measurement of distance between MF and AMF. The origin (O) was defined as a centre of the mental foramen and x-axis was parallel to the occlusal plane. The distance (*white line*) was measured by formula previously proposed by Naitoh et al. [14]: distance = $\sqrt{x^2+{y}^2}$. *White arrowhead*- mental foramen, *blue arrowhead*- accessory mental foramen

All CBCT images were obtained using a GX CB-500 (Gendex, USA) at 120 kVp and 5.0 mA, with a voxel size 0.125–0.25 mm and an exposure time of 20 s. All images were analyzed using specialized computer software (iCATVision Q, ver. 1.9.3.13; Gendex, USA). The samples were manually evaluated by independent two observers and any disagreement between them was discussed until a consensus was reached. To test the reproducibility, the two observers re-examined 50 randomly selected CBCT scans 4 weeks after the first evaluation. For the final analysis, each measurement was performed twice. Finally, obtained data by both investigators were averaged and mean values were calculated. Data were assessed on axial, sagittal and coronal CBCT slices of 0,13 mm thickness. The CBCT scans were evaluated in terms of the presence of accessory mental foramen (Figs. 1 and 2). When the AMF was present, the location and side (right or left branch of the mandible) were recorded. With regard to location, AMFs were classified into two groups according to Naitoh et al. [14]: anterior or posterior to mental foramen. The distance between the accessory mental foramen and the mental foramen was measured by formula previously proposed by Naitoh et al. [14]: distance = $\sqrt{x^2+{y}^2}$ (Fig. 3). The mean vertical size of MF on the side with and without AMF was also calculated. The obtained variables were statistically analyzed using Statistica 12.5 PL® software (StatSoft, Poland). The chi-square test was used to determine potential differences between the presence of AMFs and sex of the patients and the Mann–Whitney *U* test was used to evaluate the relationship between the vertical size of the MF and the presence of AMFs. The level of statistical significance was set at *p* < 0.05.

## Results

There were 105 males (52.5%) and 95 females (47.5%) in the study group. The average age of the 200 patients who were included in this study was 54.57 years (range: 29 to 76 years, SD: 10.26 years). The mean age of the males was 54.49 years (range: 29 – 76 years, SD: 10.79), while the mean age of the females was 54.66 years (range: 39 – 75 years, SD: 9.72).

**Fig. 4:**
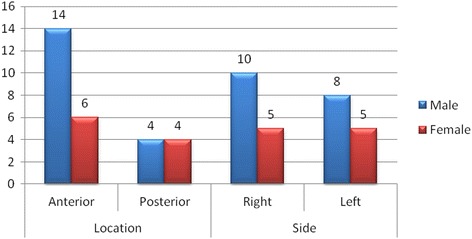
Location and side of accessory mental foramen

Twenty-eight AMFs (7%) were observed from 400 sides of 200 patients. AMFs occurred more often in males (18 AMFs) than in females (10 AMFs). No statistical significant difference was found between the occurrence of AMF and sex (*p* > 0.05). The bilateral presence of AMF was not observed in the presented study. Twenty AMFs (71.4%) were located anteriorly, and eight (28.6%) - posteriorly. Fifteen AMFs (53.6%) were on the right side and thirteen (46.4%) were on the left. The location (anterior/posterior) and side (right/left) of AMFs in respect to patient sex were presented in Fig. 4.

The distance between the AMF and the MF ranged from 0.64 to 6.5 mm with the mean of 2.86 mm (SD: 1.34 mm).

The mean values of vertical size of the MF with AMF (on the same side) and without AMF were presented in Table 1. The relationship between the mean vertical size of MF and the presence of AMF was not statistically significant (*p* > 0.05).

**Table 1:** Vertical sizes of MF and the presence of AMFs

Vertical size of MF (mm)				
	Mean	Range	SD	*P*-value
MF on the side with AMF	3.21	1.74–5.82	0.98	*p* > 0.05
MF on the side without AMF	3.26	1.12–7.02	0.97	

## Discussion

The presence of an AMF has been suggested to result from the branching of the mental nerve before it exits the mental foramen. Ignoring the presence of AMF may cause unexpected damage to the neurovascular bundles or lead to the failure of a mental nerve block.

According to Balcioglu and Kocaelli [5], the presence of AMF is a rather rare anatomical variation with the prevalence ranging from 1.4 to 10%. The differences may be explained by different imaging techniques and race. Panoramic radiography provides a flat image of the curved structure and is not as accurate as CBCT in the horizontal localization of objects. Their report also revealed that non-Caucasians have a higher prevalence of AMF than Caucasians [5]. On the other hand, accessory mental foramen in comparison to very rare skeletal variations like double suprascapular foramen are a common anomaly [21].

The prevalence of AMF varies in different ethnic groups. The highest is reported in black and Maori males [22]. Kalender et al. [23] observed AMF in 6.5% of a Turkish population, and prevalence of 6.68 and 7% were found in Greek [24] and Japanese [15] populations, respectively. In adult Sri Lankan [25] and Indian [26] populations, the prevalence of AMF was found to be 3.92 and 8.9%, respectively. In the present study, the AMFs were detected in 7% of the selected Polish population.

In our study, AMFs occurred more often in males (18 AMFs) than in females (10 AMFs). However, the difference was no statistically significant (*p* > 0,05). Other studies also confirmed that AMFs were more commonly found in males [14, 15]. Göregen et al. [8] in their study observed the same number of AMFs in males and females with no statistical differences between these groups.

The location of AMF is also important and can directly affect the treatment plan since it might interfere with performed procedures. Katakami et al. [13] in the study of 150 patients, observed the presence of 17 accessory mental foramina by CBCT with 59% of which being posteriorly located to the mental foramen. Another study conducted on 157 patients demonstrated the presence of 15 accessory mental foramina, nine of which posteriorly located [15]. On the other hand, the study of 315 patients of a Turkish population revealed the occurrence of 22 AMFs, twelve of which (54,5%) anteriorly located [8]. In the present study, twenty (71.4%) of twenty-eight AMFs were anteriorly located.

The next measured parameter was the distance between the mental foramen and the accessory mental foramen. Göregen et al. [8] reported that the distance ranged from 1.6 to 4.9 mm, with the mean of 2.54 mm (SD: 1.1 mm). In the studies of Naitoh et al. [15] and Kalender et al. [23], the mean value ranged from 4.5 to 9.6 mm, with the mean of 6.3 mm (SD: 1.5 mm) and from 1.3 to 15.4 mm, with the mean of 5.2 mm (SD: 4.4 mm), respectively. In the present study, the distance between the AMF and the MF ranged from 0.64 to 6.5 mm with the mean of 2.86 mm (SD: 1.34 mm)

The mean vertical size of the MF on the same side as the AMF was 3.21 mm (range: 1.74 – 5.82 mm, SD: 0.98), and that of MF on the sides without AMF was 3.26 mm (range: 1.12 – 7.02 mm, SD: 0.97 mm). The relationship between the mean vertical size of the MF and the presence of the AMF was not statistically significant (*p* > 0.05). Similar results were reported by Naitoh et al. [15] and Göregen et al. [8].

In 2013, Chen et al. [27] described a new method of improvement of computed tomography images. They used fast dictionary learning-based processing. This method brings encouraging improvements in abdomen low-dose computed tomography images with tumours. In 2014, Chen et al. [28] used novel image-domain algorithm called "artefact-suppressed dictionary learning". In this method, orientation and scale information on artefacts is exploited to train artefact atoms. These artefact atoms are then combined with tissue feature atoms to build three discriminative dictionaries. Authors provided qualitative and quantitative evaluations of this method on a large set of abdominal and mediastinal computed tomography investigation. In 2016, Chen et al. [29] described new technique of minimal path propagation with backtracking for curve-like structure extraction. They found that the information in the process of backtracking from reached points can be well utilized to improve the extraction performance.

Preoperative imaging study is important prior to any surgical or anaesthetic procedure in mental regio. AMF is a relevant anatomic structure, which should be considered in treatment plan of procedure performed in mandible (i.e. root resection of mandibular premolars or molars, osteotomy, mandibular rehabilitation after trauma, placement of dental implants). The detection of AMF has a direct influence on therapeutic success. In patients with AMF, accessory mental nerve and vessels may be present. The presence of accessory innervations may explain failures to achieve adequate levels of anaesthesia during surgical and routine dental procedures using conventional nerve block techniques. The detection of AMF can prevent nerve and vascular injury and reduce complications of dental treatment such as: paralysis, hemorrhage and post-operative pain.

## Conclusion

In the present study, the occurrence of the accessory mental foramen in the study of the Polish population is similar to that described in Turkish, Greek and Japanese population. Awareness of anatomy and its variations are essential to ensure precise dental procedure execution. Moreover, it is crucial to better understand possible anatomical conditions that can promote neurosensory disturbance in treated area.

## Abbreviations

AMF: Accessory mental foramen
CBCT: Cone-beam computed tomography
MF: Mental foramen
